# Supplementary material for: Image-Guided Precision Medicine in the Diagnosis and Treatment of Pheochromocytomas and Paragangliomas
Source: Cancers (Basel). 2023 Sep 21;15(18):4666. doi: 10.3390/cancers15184666 (PMC10526298; doi:10.3390/cancers15184666)
Supplement: Supplementary file 1 [file cancers-15-04666-s001.zip › cancers-2335151-supplementary.pdf]

Supplementary material

Table S1. Metabolic imaging radiotracer used for imaging techniques.

	Name	Advantage	Mechanisms of action	Performances	Disadvantages, adverse events
**SSTa**	^{68}Ga-DOTA-TATE	- all SSTa are theranostic tracers<br>- more accurate in lesions detection after negative MIBG scintigraphy in PPGLs[106,220,221]	^{68}Ga-labeled DOTA-conjugated somatostatin analogues	90-98% Se	Lack of availability worldwide<br>Need for expensive generator and experienced radiopharmacy unit
	^{68}Ga-DOTA-NOC	-detection of NETs metastasis<br>-more efficient in comparison to ^{18}F-DOPA [222]	good affinity for SSR-2, SSR-3 and SSR-5[223]	100% Se and Spe[224]	
	^{68}Ga-DOTATOC	-good sensibility for metastatic PCCs<br>-detection of NETs bone metastases [225]		(92%)[226]	
	^{68}Ga-DATATOC	easier and quicker preparation of the radiopharmaceutical tracer than DOTA. [227]		to be determined	
**MIBG**	^{123} or ^{131}I-MIBG	Theranostic tracer<br>Long experience<br>Better analysis than CT/MRI when anatomical distorsion (post-surgery, scars, metallic clips)	Analogue of guanethidine, cellular uptake by NET	Se 74-97% in PPGL (lower if metastatic) except thoracic and HaN PGL : poor	Physiological uptake in adrenal gland<br>Drug interactions
**FDA/FDOPA**		Low uptake in normal adrenal gland	FDOPA is a precursor of FDA in catecholamine synthesis, cellular uptake by LAT1, decarboxylated into FDA	Se and Spe superior to 90%	Lower sensibility in MPPGLs and SDHx-related PPGLs<br>Not a theranostic tracer
**FDG**		poor sensitivity for non-metastatic sporadic PCCs (58%)	FDG is a surrogate of glucose, with a cellular uptake mainly by GLUT1 and GLUT 3 transporters	Better than MIBG scintigraphy, in MPPGLs (82%)<br>High sensitivity in SDHx patients	Lack of specificity for PPGL and PCC<br>Not a theranostic tracer

Table S2. Systemic and theranostic therapies.

	Target	Cellular clinical effects	Radiological patterns of response	Clinical benefits, ongoing trials
**Chemotherapy**	CVD-protocol: cyclophosphamide, vincristine and dacarbazine	catecholamine excretion reduction?	decrease in size	- catecholamine excess improvement (30-40% of patients) -unclear benefit on overall survival in MPPGLs for several adverse events[147–149] LAMPARA trial (NCT03946527), assessing Lanreotide in MPPGLs
**SSTa therapies**		tumor cells growth inhibition apoptosis activation		significant improvement of clinical symptoms (flush, diarrhea) in GEP NETs[161,162]
**Interferon alpha**	natural killer lymphocyte functions stimulation		growth control (stable disease)	clinical improvement of pain, headaches, paradoxical diarrhea, sweating
**Tyrosine kinase inhibitors (TKI)**		anti-neoangiogenic, pro-apoptotic, inhibitor of cell growth and cell migration	decrease in size[158,163] diminution of glucose uptake[159] PFS improvement[160]	- Sunitinib : FIRST-MAPPP trial NCT01371201 - Axitinib: NCT01967576 - Cabozantinib: NCT02302833
**Immune checkpoint inhibitors (ICI)**	- CTLA-4: ipilimumab - PD-1: nivolumab, pembrolizumab - PD-L1: aterolizumab, avelumab, durvalumab	- tumor reduction - hormonal secretion reduction in MMPPGLs	pseudoprogression hyperprogression abscopal effect	NCT02721732: rare tumors including MPPGLs
**PRRT**	^{90}Y-DOTATOC	limited adverse events (nausea, reversible hematopoietic toxicity)[182,183]	-good sensibility for metastatic PCCs - detection of NETs bone metastases [225] - morphological response[181]	symptomatic response (pain, carcinoid syndrome)
	^{177}Lu-DOTATATE	(^{177}Lu-DOTATATE in mPPGLs is promising but should be limited to MIBG-negative patients[228]) Encouraging preliminary studies hint at similar results as	RECIST 1.1 criteria are widely used for studies.	^{177}Lu is not a pure beta-emitter, but also emits low energy gamma-rays, allowing post-therapy imaging and dosimetry ongoing clinical trial (NCT03206060) PRRT in inoperable PPGL

		those obtained for NETs (ref)		(^{177}LuDOTATE for PCCs and ^{68}GaDOTATATE for PGLs)
**Iobenguane or ^{131}I-MIBG**[174–176]			reduction in size on CT scan, and a lower MIBG uptake[177]<br>In HaN PGLs, potential alternative when neurovascular structure location is a contraindication to surgery or radiation therapy, to achieve PR or SD[187]	β- particle and γ-radiation by nuclear transformation

Table S3. Imaging biomarkers currently used in PPGLs.

		**Imaging modality/ calculation method**	**Advantage**
**Anatomical imaging**	Tumor Growth Rate TGR	2D on CT scan, as the percentage change in tumor size over one month (%/m)	early predictor of PFS [203,204]
	Volume assessments for early detection of change	3D on CT scan, volume measurement	better inter-operator agreement,<br>earlier partial response or progressive disease[202]
**SPECT / PET metabolic biomarkers**	SUV max	2D on PET CT	- in advanced (NSCLC)significantly predictive of response to anti-PD-1 antibody<br>-from pretherapeutic ^{68}Ga-DOTATOC-PET/CT for NET, predictive of response probability of PRRT.therapy[229,230]
	Metabolic Tumor Volume (MTV)	a margin threshold of x% of SUVmax	pancreatic NETs: predictor of overall survival[231]
	Total Metabolic Tumor Volume (TMTV)	3D	In advanced NSCLC or melanoma treated with ICIs : high pretreatment ^{18}F-FDG PET TMTV was associated with shorter PFS<br>-risk stratify patients with different overall survival probabilities[232,233]

	Somatostatin Receptor Expressing Tumor Volume (SRETV, mL) and Total Lesion Somatostatin Receptor Expression (TLSRE, g)	^{68}Ga-DOTATATE PET/CT, 3D	significant and independent prognostic value in well-differentiated NETs on PFS[176]
**Radiomics**	Asphericity (ASP)	^{111}In-octreotide scintigraphy prior to PRRT imaging-based quantification of lesion's spatial heterogeneity with an automatic algorithm for delineation	GEP-NETs response prediction : higher ASP was significantly associated with worse response [234]
	Textural features (TF):	2D and 3D, voxel level entropy, homogeneity, intensity variation…	In GEP-NET patients undergoing pre-therapeutic SSTR-PET CT, entropy and intensity were significant predictors of OS[177].
